# Investigation of Intramolecular Dynamics and Conformations of α-, β- and γ-Synuclein

**DOI:** 10.1371/journal.pone.0086983

**Published:** 2014-01-28

**Authors:** Vanessa C. Ducas, Elizabeth Rhoades

**Affiliations:** 1 Department of Molecular Biophysics and Biochemistry, Yale University, New Haven, Connecticut, United States of America; 2 Department of Physics, Yale University, New Haven, Connecticut, United States of America; University of Florida, United States of America

## Abstract

The synucleins are a family of natively unstructured proteins consisting of α-, β-, and γ-synuclein which are primarily expressed in neurons. They have been linked to a wide variety of pathologies, including neurological disorders, such as Parkinson’s disease (α-synuclein) and dementia with Lewy bodies (α- and β-synuclein), as well as various types of cancers (γ-synuclein). Self-association is a key pathological feature of many of these disorders, with α-synuclein having the highest propensity to form aggregates, while β-synuclein is the least prone. Here, we used a combination of fluorescence correlation spectroscopy and single molecule Förster resonance energy transfer to compare the intrinsic dynamics of different regions of all three synuclein proteins to investigate any correlation with putative functional or dysfunctional interactions. Despite a relatively high degree of sequence homology, we find that individual regions sample a broad range of diffusion coefficients, differing by almost a factor of four. At low pH, a condition that accelerates aggregation of α-synuclein, on average smaller diffusion coefficients are measured, supporting a hypothesis that slower intrachain dynamics may be correlated with self-association. Moreover, there is a surprising inverse correlation between dynamics and bulkiness of the segments. Aside from this observation, we could not discern any clear relationship between the physico-chemical properties of the constructs and their intrinsic dynamics. This work suggests that while protein dynamics may play a role in modulating self-association or interactions with other binding partners, other factors, particularly the local cellular environment, may be more important.

## Introduction

The synuclein proteins are small (∼14 kDa), soluble proteins expressed predominantly in neurons, although their cellular localization varies. Both α-synuclein (αS) and β-synuclein (βS) are found ubiquitously in pre-synaptic terminals throughout the brain, while γ-synuclein (γS) tends to be localized in more differentiated nerve cells, and away from the forebrain [1], [2]. The synucleins have also been found in various other cell types including liver (γS), skeletal muscle (αS, βS, and γS), lung (αS) and red blood (αS) cells [1], [3].

**Figure 1:**
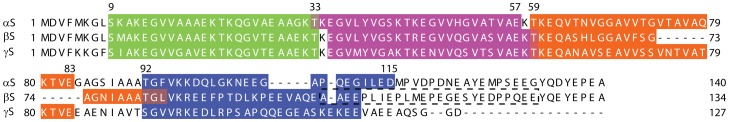
Synuclein proteins sequences. The various regions probed here are indicated by the colored boxes: green – AH construct (88% identity between αS and βS and 80% identity between αS and γS); purple – LF construct(74% identity between αS and βS and 59% identity between αS and γS); orange – NAC construct (48% identity between αS and βS and 41% identity between αS and γS); and blue – CT construct (27% identity between αS and βS and 25% identity between αS and γS). The sequence enclosed by the dashed line corresponds to an additional proline-rich construct (residues 102 to 126) probed in the C-terminal region of βS.

The synucleins have generally been described as intrinsically disordered proteins (IDPs) although recent studies suggest that at least αS might exist in a partially structured, oligomeric state under some conditions [3]–[5]. Their primary sequence can be divided into two segments: 1) a N-terminal region rich in basic amino acid residues that mediates binding to membranes, and 2) a net negatively-charged C-terminal region that has no well-established function, but that might be involved in protein-protein interactions as well as in inhibiting aggregation [6], [7]. Further sequence analyses of the synucleins indicate the presence of several repeat motifs in their N-terminal regions, displaying variations of the consensus sequence KTKEGV, that share significant similarities with lipid-binding motifs found in apolipoproteins; αS and γS each have seven repeat motifs, while βS has six [8]–[10]. Other sequence differences include the central region of βS which lacks a stretch of 11 amino acids, corresponding to residues 73 to 83 of the non-amyloid beta component (NAC) region of αS (Figure 1) [1], [11]. Furthermore, γS has a shorter, slightly less negative, C-terminal region. These sequence variations may be responsible for the proposed differing native functions of the synucleins.

αS is the most well-studied of the synucleins, primarily due to its link to Parkinson’s disease (PD). It is the major constitutive component of Lewy bodies, cytoplasmic inclusions that are hallmark of the disease [12]–[14]. Five familial mutations, A30P, E46K, H50Q, G51D, and A53T, as well as multiplication of the αS gene, have been linked to early-onset PD [12], [15]–[18]. Aggregates of αS are also found in numerous other neurological disorders such as dementia with Lewy bodies, multiple system atrophy, Alzheimer’s disease, the parkinsonism-dementia complex of Guam, and pantothenate kinase-associated neurodegeneration (formerly known as Hallervorden-Spatz disease) [19]. Despite their similarities to αS, significantly less is known about βS and γS. Recent investigations have also linked mutations in βS, V70M and P123H, to cases of dementia with Lewy bodies [20]. Furthermore, work from our lab found that these βS variants bind model membranes more tightly than the wild-type protein [21]. The observed increase in lipid-binding affinity for both mutants may be relevant to their noted ability to enhance synucleopathies and to their reported toxic gain of function manifested by a greater propensity to self-aggregate both in vivo and in vitro [22]–[24]. Alternatively, wild-type βS in the absence of lipid bilayers has an inhibitory effect on αS aggregation in vitro, a property that may be exploitable for the development of therapeutics [25].

In contrast to αS and βS, γS has been linked to various cancers. Specifically, it has been shown that γS is capable of conferring drug-resistance to cancerous cells and of promoting metastasis in breast carcinomas [26]–[29]. Recent simulations suggest that interactions with the proteins involved in those processes are mediated by the C-terminal domain of γS [30]. Upregulation of γS has also been associated with several ocular diseases and increased synucleopathies resulting from its aggregation and accumulation into cellular lesions [31]–[34].

The monomeric synucleins do not form stable secondary or tertiary structures in solution, although varying degrees of structural propensity are present in some domains [35], [36]. Structural propensity is expected to correlate with intrachain dynamics, where highly flexible domains are less likely to adopt stable conformations that foster bimolecular interactions compared to relatively stiffer domains. Moreover, lack of stable structure does not necessarily imply that the structures are completely random. The ‘unfolding’ of IDPs, similar to the misfolding of globular proteins, is known to promote aggregation-prone protein conformations [37]–[39]. However, as highlighted by the varying results of in vitro fibrillation assays with the synuclein proteins, not all IDPs are readily fibrillogenic [7], [36], [40].

Here, we seek to determine how the intrinsic dynamics of all three synuclein proteins relate to their requirement for mediating interactions with binding partners relevant to function, as well as their propensity to self-associate relevant to disease. To do so, we use fluorescence correlation spectroscopy (FCS) and single molecule Förster resonance energy transfer (FRET) to measure the intrachain diffusion coefficients of four analogous regions within αS, βS, and γS.

## Materials and Methods

### Expression and Purification of Recombinant Synucleins

βS and γS were cloned as N-terminally His-tagged constructs with a tobacco etch virus (TEV) cleavage site separating the purification tag from the protein sequence; all three proteins were expressed from E. coli as described previously [41], [42]. Briefly, BL21 cells were transformed with the plasmid of interest and grown in ampicillin-supplemented LB media until the OD_600_ reached 0.5–0.6. Protein expression was then induced with the addition of 400 µM isopropyl-β-thiogalactoside (IPTG). The cell lysates of the His-tagged constructs were incubated with Ni-NTA agarose resin (Qiagen) and then washed with increasing concentrations of imidazole to elute the proteins. The His-tag was removed by incubation with TEV protease at a 2∶1 protein-to-protease ratio at room temperature overnight. Cleaved proteins were separated from uncleaved proteins by a second incubation with the Ni-NTA resin. The cleaved protein was collected in the flow-through of the column and was separated from remaining contaminants on a Superdex 75 size-exclusion column (GE Healthcare). Growth and expression of αS followed a similar protocol to that of βS and γS. αS cell lysate was centrifuged at 16900×g and the supernatant was subjected to two ammonium sulfate cuts, αS precipitating in the second cut. The pellet was resuspended in anion-exchange running buffer (25 mM Tris pH 8, 20 mM NaCl, 1 mM EDTA), treated with 1 mM phenylmethylsulfonyl fluoride (PMSF), and dialyzed overnight at 4°C against the same running buffer. The protein sample was syringe-filtered, applied to an anion-exchange HiTrap Q FF column (GE Healthcare) and eluted with a NaCl gradient. Collected fractions were pooled and run through a Superdex 75 size-exclusion column (GE Healthcare) for further purification from remaining contaminants. The recombinant γS used in this work has a valine residue at position 110 although a common polymorphism of γS (V110E) has been reported previously [43]. The degree of homology among the individual domains used in this study was determined using Clustal Omega and the sequence alignment was done with Jalview [44], [45]. For site-specific labeling, cysteine residues were introduced at desired positions (Figure 1) in all three proteins using a protocol based on the QuikChange mutagenesis kit (Stratagene).

### Labeling of Protein Constructs

Approximately 350–400 µL of 100 µM protein were used for the tetramethylrhodamine (TMR) labeling reaction. The protein samples were incubated with 1 mM TCEP, stirring at room temperature for 5 minutes. 10 µL of ∼1 mg/mL TMR in anhydrous DMSO were added to the protein samples dropwise, while stirring, and incubated at room temperature for 2 hours or overnight at 4°C. The labeled samples were first run over two HiTrap desalting columns (GE Healthcare) mounted in series. The columns were first washed with 10 mL of working buffer (50 mM Tris pH 7.4, 100 mM NaCl) before loading the labeled sample. The labeled protein was eluted in the first 1 mL following the void volume (3 mL), using 10 mL of the working buffer. The eluted labeled protein was then run over a size-exclusion column for an additional purification step. The labeled protein fractions were pooled, buffer-exchanged from the size-exclusion column running buffer (25 mM Tris pH 8.0, 100 mM NaCl, 1 mM EDTA, 0.5 mM TCEP) into the working buffer described above, and concentrated to approximately 250–500 µL. The concentration of the labeled samples was determined using a modified Lowry assay, with the corresponding unlabeled protein constructs serving as standards. For single molecule FRET measurements, ∼200 µL of 100 µM protein were incubated at room temperature with 1 mM dithiothreitol (DTT) for 30 minutes. The DTT was removed using the HiTrap desalting columns. The acceptor (Alexa 594 maleimide) and the donor (Alexa 488 maleimide) dyes were added sequentially to the protein sample at a 5∶1 (acceptor:donor) ratio and incubated at room temperature for 2 hours. Unconjugated dye was removed using the coupled HiTrap desalting columns.

### TMR-quenching Measurements and Analysis

FCS measurements of protein labeled with TMR were collected on a lab-built instrument based around an inverted Olympus IX71 microscope using a 561 nm wavelength laser as the excitation source. The output power from the laser was adjusted to ∼40 µW just before the side-port entry of the microscope using neutral density filters. The fluorescent signals were collected through the objective and separated from the laser with a 565DCXR dichroic and a HQ580LP emission filters (Chroma). The emitted signal were further divided by a 50/50 beamsplitter and focused to the apertures of two 50-micron aperture optical fibers (OzOptics), each coupled to an avalanche photodiode (Perkin Elmer). A digital correlator (Flex03LQ-12, correlator.com) was used to obtain pseudo-crosscorrelation curves. Crosscorrelation of the fluorescence signal eliminates the afterpulsing signal in the detectors and allows for resolution of faster timescales in the resulting curves [46].

**Figure 2:**
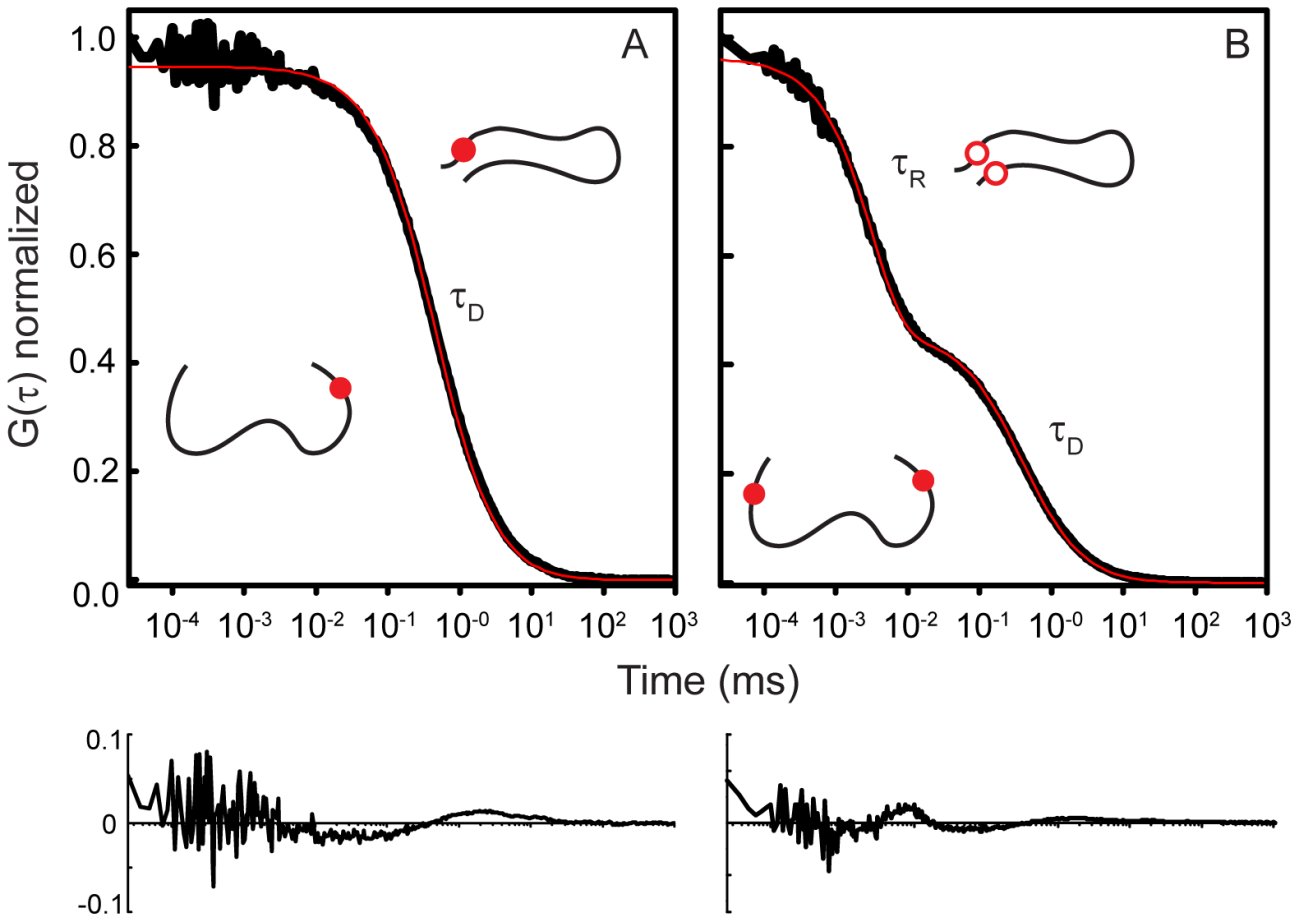
Representative autocorrelation curves of a single-labeled control construct (A) and of a double-labeled construct (B). (A)The control autocorrelation curve shows the characteristic decay curve observed for the simple translational diffusion (τ_D_) with no evidence for a second decay component. The TMR remains fluorescent (solid red circle), independent of the conformation of the protein. (B)The autocorrelation curve of the double-labeled construct has an additional fast decay component (τ_R_), reflecting the intrachain diffusion that brings the fluorophores into close proximity, resulting in self-quenching (open red circles), which is relieved when the fluorophores diffuse apart (solid red circles). Red traces correspond to fits with Eq. 1 where A = 0 (no kinetic component; A) and with Eq. 1 (B), with accompanying residual plots.

All measurements were made in eight-well Nunc chambers (ThermoScientific) passivated with polylysine-conjugated polyethylene glycol to prevent protein adsorption to chamber surfaces. Measurements were made at room temperature (20.5±0.1°C) in Tris buffer pH 7.4 or 3.5 (50 mM Tris 100 mM NaCl, 2 mM TCEP). Before each measurement, 50 nM of the TMR-labeled protein was incubated with TCEP to avoid cysteine-mediated dimer formation. The samples were excited with a laser power adjusted to 35–40 µW. For a single measurement, ten to fifteen autocorrelation curves, of 30 seconds each, were collected (Figure 2). Each set of curves was averaged and the average curve was fit as described below, using the inverse of the variance in the curves as a weighting factor. The reported τ_R_ are from at least four measurements (averaged curves derived from ten to fifteen individual curves) of at least two different sample preparations collected under similar conditions. Control measurements conducted at different laser powers indicated that the observed τ_R_ values are not due to triplet-state photophysics (Figure S1).

FCS analyzes the fluctuations in the fluorescence generated by molecules within a well-defined observation volume. The autocorrelation of the fluorescence signal yields information about the timescales of the processes resulting in the fluorescence fluctuations, including translational diffusion, chemical reactions, and photophysical processes. Here, we use FCS to measure conformational dynamics of proteins site-specifically labeled at two positions with TMR by autocorrelation of the fluorescence fluctuations resulting from self-quenching of the fluorophores (Figure 2B) [47]–[49]. When the two TMR moieties are within a close range of one another, the dye molecules can stack and interact via van der Waals attractive forces to form a dimer, which results in mutual fluorescence quenching. When the fluorophores diffuse away from each other, the self-quenching is relieved and the TMR molecules are once again fluorescent. The resulting autocorrelation curve reflects changes in fluorescence (Eq. 1) due to both the translational diffusion (τ_D_) of proteins through the observation volume, as well as self-quenching of the fluorophores arising from intrachain diffusion (τ_R_) while the molecules are within the observation volume.
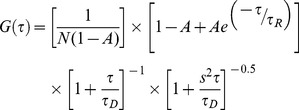
(1)


Additional parameters are: N, number of molecules in the observation volume; s, ratio of the radial to axial dimensions of the observation volume; A, amplitude of the fast component of the curve, corresponding to the average fraction of molecules in the quenched state. s, was determined as a floating parameter from measurements of Alexa 594 hydrazide (s = 0.175) and fixed for all subsequent fitting. Control measurements made of proteins labeled with a single TMR report only on the diffusion of the protein and are fit by Eq. 1 where A = 0 (Figure 2A). No significant difference in the τ_R_ values were found when two kinetic components were used in fitting the double-labeled constructs (Eq. S1 and Eq. S2; Figure S2).

### FRET Measurements and Analysis

Single molecule FRET measurements were taken using the same lab-built instrument described above, using a 488 nm wavelength laser as the excitation source, and appropriate emission filters as described previously [50]. The energy transfer efficiency (ET_eff_) between the donor-acceptor pair was determined using Eq. 2, where I_A_ is the signal collected in the acceptor channel; I_D_, the signal collected in the donor channel. β and γ are correction factors that have been determined for the setup used in our laboratory: β (0.6) corrects for the bleed-through of donor fluorescence into the acceptor channel, while γ (1.2) corrects for the variations in quantum yield between the donor and acceptor fluorophores and for detection efficiency [50].
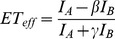
(2)


**Figure 3:**
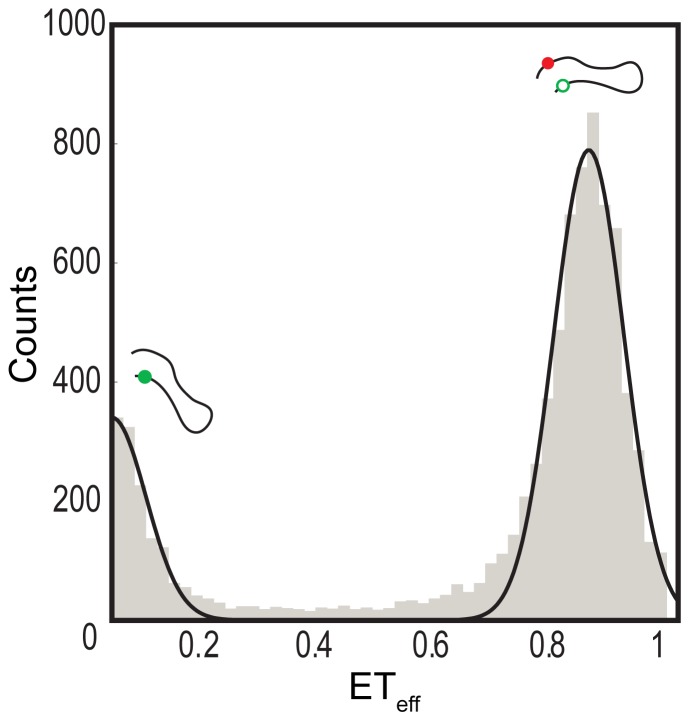
Representative single-molecule FRET histogram of the CT construct of αS. The major peak at ET_eff_∼0.84 arises from energy transfer between residues 92 and 115, while the peak at ET_eff_∼0 results from donor-only molecules.

A threshold value was determined and applied during data processing to isolate background signals from FRET signals generated by the double-labeled protein molecules. To select the threshold, signals were measured for buffer alone and then compared with signals collected with double-labeled protein. The values obtained from Eq. 2 were used to construct histograms (Figure 3), which were then fit to determine the mean ET_eff_ value for the given construct using a double-Gaussian equation (Eq. 3), fitting both the zero (donor only) and the data peaks (Origin).
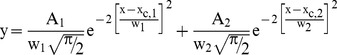
(3)Where A is the area; w is the width of the peak and x_c_ is the center of the peak, corresponding to the mean ET_eff_. Thousands of events were collected on average and used to construct the FRET efficiency transfer histograms from which mean ET_eff_ values were extracted. For each construct, a minimum of three histograms derived from a minimum of two different sample preparations were fit.

### Statistical Analysis

All results are reported as mean ± standard deviation (σ) from at least three sets of measurements made on at least two different sample preparations. The diffusion coefficient (D) values were calculated from the observed relaxation times and the radii of gyration (R_g_) determined from the mean ET_eff_ values (File S1, Eq. S3, and Table S5). The error in R_g_^3^, defined as δ_Rg3_ = [R_g_^3^⋅3⋅(σ_Rg_\R_g_)], and the error in τ_R_ (standard deviation of the mean, Table 1) were propagated in determining the error in D, calculated as δ_D_ = [(3.545/a) ⋅(δ_Rg3/τR_)], where a is the contact distance between TMR dyes resulting in quenching (File S1). Statistical significance was evaluated using a two-tailed Mann-Whitney U-test with a critical significance level (α) of 0.05.

**Table 1:** Intrachain dynamics at pH 7.4 and pH 3.5, reported as observed relaxation time, τ_R_ (µs).

	αS		βS		γS	
Construct	pH 7.4	pH 3.5	pH 7.4	pH 3.5	pH 7.4	pH 3.5
AH	2.08±0.47	2.35±0.19	1.96±0.13	2.42±0.42^+^	2.41±0.30	1.36±0.39*^;+^
LF	1.10±0.11	1.20±0.18	1.16±0.08	1.81±0.07*^;+^	1.14±0.11	1.05±0.06
NAC	1.23±0.07	1.48±0.05^+^	0.84±0.03*	1.59±0.09^+^	1.72±0.24*	1.40±0.20*^;+^
CT	1.37±0.08	2.44±0.35^+^	1.81±0.07*	2.50±0.20^+^	1.14±0.05*	0.98±0.05*^;+^

Observed intrachain diffusion time obtained from FCS measurements as described in the Materials and Methods (4≤n≤12 for all constructs; error is standard deviation of the mean). Significance level in the difference in dynamics of βS or γS relative to αS (*); significance level in the difference in dynamics for each protein at pH 3.5 relative to pH 7.4 (+). */+ indicates results that are significant at the 5% level using a two-tailed Mann-Whitney U-test. AH–amphipathic helix motif-containing construct; LF–flexible loop forming construct; NAC–non-amyloid beta component or hydrophobic core construct; CT–C-terminal construct.

## Results

We selected four regions (∼25 residues each) within the synuclein proteins allowing for comparison of dynamics of different domains within each protein as well as between proteins (Figure 1). For each construct, end-to-end contact rates were measured by FCS and the root-mean-square (RMS) distances were determined from single molecule FRET. Each construct probes a particular region of interest of the protein: 1) residues 9 to 33, containing two amphipathic helix motifs (AH); 2) residues 33 to 57 in αS or 35 to 59 in βS and γS, include the highly flexible loop-forming (LF) region that links the two α-helical domains observed on detergent micelles [51]; 3) residues 59 to 83, corresponding to the hydrophobic core or non-amyloid beta component domain of αS (NAC); and 4) residues 92 to 115 in αS, 81 to 105 in βS, and 92 to 117 in γS, probing part of the negatively-charged C-terminus (CT) of each protein. All measurements were taken with full-length proteins modified to introduce cysteine mutations that delimit the region of interest. For example, residues S9 and T33 in the wild-type proteins were changed into cysteine residues, which were then used for site-specific labeling. This approach allowed us to monitor the properties of the segments within the full-length protein.

### The AH Regions have Slowest Dynamics

**Figure 4:**
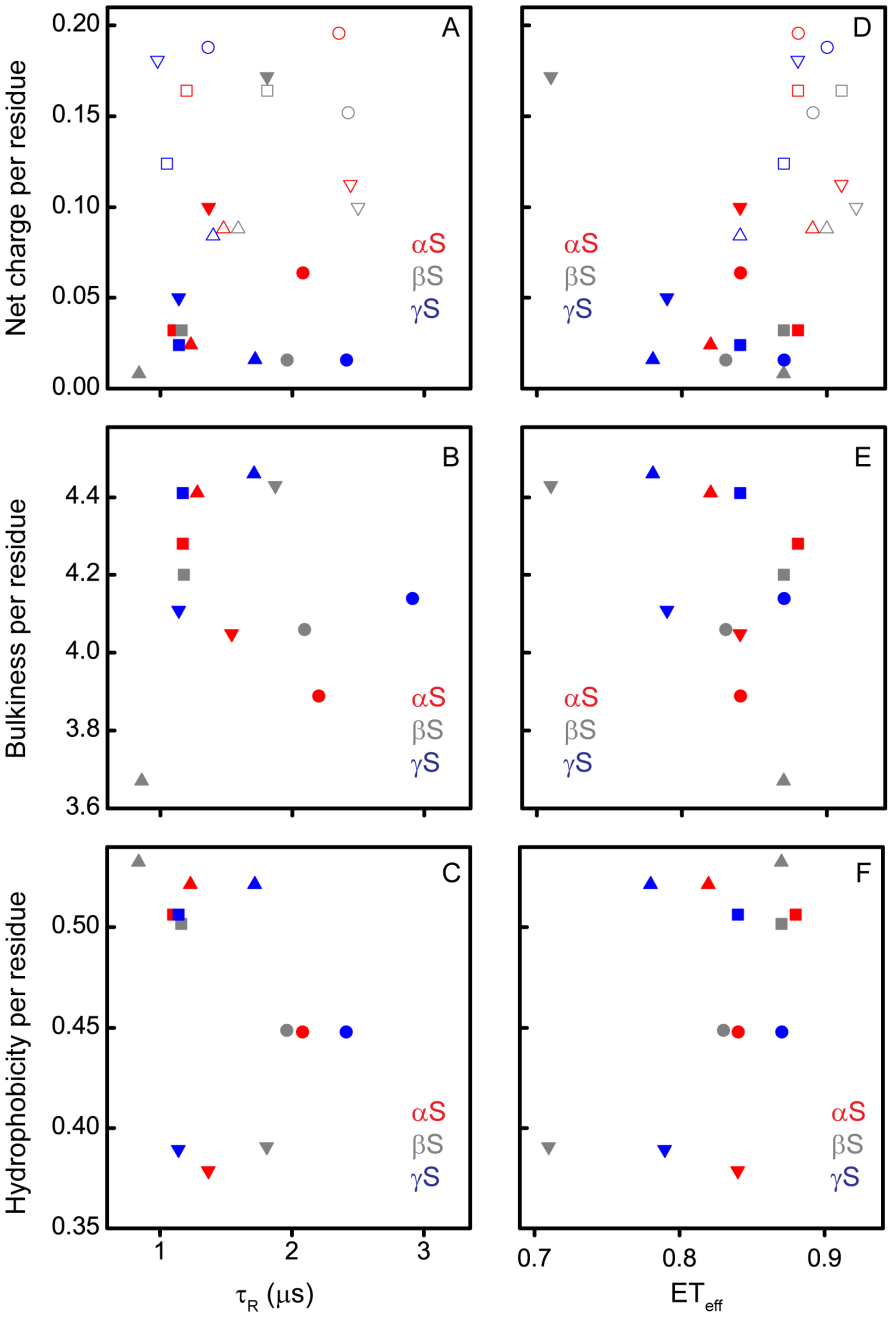
τ_R_ and Mean ET_eff_ as a function of the physico-chemical properties of the synucleins. Net charge per residue (A and D), bulkiness per residue (B and E) and hydrophobicity per residue (C and F) at pH 7.4 (solid symbols) and/or at pH 3.5 (open symbols). Circles– AH constructs; squares – LF constructs; triangles – NAC constructs; inverted triangles – CT constructs.

Using FCS, we monitored the extent of flexibility within the regions being probed by determining the time parameter (τ_R_) that characterized the self-quenching reaction of TMR. The resulting autocorrelation curves of the diffusing double-labeled constructs show two components, a fast component of a few microseconds corresponding to the TMR quenching reaction and a slower component in the low millisecond range, which corresponds to the translational diffusion of the protein (Figure 2B). The end-to-end contact time of the TMR moieties is expected to correlate with the flexibility of the protein sequence within the two positions of the TMR labels, with flexible segments having relatively faster contact time than less flexible segments. First, we measured the end-to-end contact times at physiological pH (pH 7.4), where the proteins exhibit mostly random coil characteristics, with some regions of residual structure [41], [52]. The results are summarized in Table 1. There were no significant differences in dynamics within the AH and LF constructs between the three proteins. These results likely reflect the fact that these regions are the most highly conserved among them. In contrast, their NAC and CT domains do show significant differences in their τ_R_ values. Notably, at the physiological pH, the NAC construct from βS demonstrates faster dynamics, ∼50% and ∼100% faster, than the comparable regions from αS and γS, respectively. Relative to the other two synucleins, βS lacks a stretch of 11-residues within that region. Calculating the ‘bulkiness’ per residue of this segment [53] indicates that βS (∼3.7) has fewer bulky amino acids than either αS or γS (∼4.4 for each) (See Table S1), which may at least partially account for the more rapid dynamics, although we note that we do not find a general direct correlation between bulkiness and dynamics (Figure 4B). Lastly, the characteristic timescale observed for the C-terminal domain of γS is ∼20% and ∼60% faster than that of αS and βS, respectively. Like the C-terminus of γS, our CT construct of γS is less negatively charged compared to the CT constructs of αS and βS. These results indicate that electrostatic repulsion in the more negatively charged constructs from αS and βS may slow their dynamics.

The constructs were also measured at pH 3.5, a condition which promotes aggregation of αS [54], [55], and increases the average positive charge of all three proteins. Both αS and βS show increase in τ_R_ for all four constructs at low pH compared to pH 7.4, although the magnitude of the change is generally larger for βS (Table 1). Conversely, lowering the pH causes a decrease in all τ_R_ values for γS (Table 1). Interestingly, at both pH values, the AH constructs, which includes part of the region most important for binding to membranes [56], [57], generally have the slowest dynamics relative to the other constructs studied.

### Solution Conformations are Relatively Compact Disordered Ensembles

Although our constructs all probe regions of comparable linear length within the proteins, sequence variation can lead to differences in their dimensions, and thus differences in the relative volume of conformational space sampled [58], [59]. To allow for normalization of dynamics based on the intrinsic dimensions of each sequence probed, we used single molecule FRET to measure the average conformational properties of each construct (Figure 3). For these measurements, the same constructs described above were labeled with a FRET donor-acceptor dye pair (See Materials and Methods for details). The mean energy transfer efficiencies (ET_eff_) values for each construct, from which the RMS distances (Table S4) and radii of gyration (R_g_; Table S5) can be determined, are reported in Table 2.

**Table 2:** Mean energy transfer efficiency values (ET_eff_) at pH 7.4 and pH 3.5.

	αS		βS		γS		
Construct	pH 7.4	pH 3.5	pH 7.4	pH 3.5	pH 7.4	pH 3.5	
AH	0.84±0	0.88±0.01^+^	0.83±0.01	0.89±0*^;+^	0.87±0.01*	0.90±0.01*^;+^	
LF	0.88±0.1	0.88±0.02	0.87±0.01	0.91±0.01*;^+^	0.84±0.01*	0.87±0^+^	
NAC	0.82±0	0.89±0.01^+^	0.87±0*	0.90±0.01^+^	0.78±0.01*	0.84±0.01*^;+^	
CT	0.84±0.01	0.91±0.01^+^	0.71±0.01*	0.92±0^+^	0.79±0.01*	0.88±0.01*^;+^	

ET_eff_ values corresponding to the mean energy transfer efficiency obtained from histograms as described in the Materials and Methods (n = 4 for all constructs except for αS LF pH 7.4 where n = 3); error is standard deviation of the mean. Significance level of βS or γS ET_eff_ values relative to αS (*); significance level in ET_eff_ for each protein at pH 3.5 relative to pH 7.4 (+). */+ indicates results that are significant at the 5% level using a two-tailed Mann-Whitney U-test. AH–amphipathic helix motif-containing construct; LF–flexible loop forming construct; NAC–non-amyloid beta component or hydrophobic core construct; CT–C-terminal construct.

At physiological pH, we observed small, but some significant, differences in mean ET_eff_ values for the regions probed in αS, βS and γS, with the greatest differences being seen with the NAC and CT domains (Table 2), thus mirroring the observations made with the TMR self-quenching measurements (Table 1). Most of the constructs return mean ET_eff_ values between 0.82 and 0.90, which convert to RMS values of ∼31 Å to 26 Å by a Gaussian chain model [60]. These more extended ensembles correspond to roughly what would be expected for an ideal random coil of ∼25 residues length which would have a corresponding RMS of ∼32 Å or 33 Å, while the others reflect a more compact disordered ensemble that has been observed previously for IDPs [59], [61], [62].

At the lower pH, we observed minor differences in the dimensions of the individual domains within each protein (Table 2), with 11 of the 12 constructs showing higher mean ET_eff_ values relative to those at neutral pH. The compaction of the CT constructs is not surprising, as the net change in charge, which has been shown to impact the dimensions of the disordered proteins [59], [63], is greatest for those constructs.

### Direct Correlation between Diffusion Coefficient and Protein Bulkiness

In order to assess whether the parameters measured correlate with any physico-chemical properties of the constructs, we plotted the net charge per residue (at pH 7.4 and 3.5), bulkiness per residue (at pH 7.4) [53], and hydrophobicity per residue (at pH 7.4) [64] of each construct (see File S1 for details of calculations; Table S1 and Table S3) as a function of τ_R_ and the mean ET_eff_ values (Figure 4). From these representations of the data, no clear general correlations can be discerned between these properties and τ_R_ (Figure 4, panels A, B, and C). The ET_eff_ plots are more revealing (Figure 4, panels D, E, and F). At pH 7.4, the data are clustered in a relatively small region of charge/ET_eff_ space (mean ET_eff_ = 0.83±0.05; Figure 4D, solid), with a shift to more compact structures at pH 3.5 (mean ET_eff_ = 0.89±0.02; Figure 4D, open) concurrent with an increase in net charge. The notable outlier to the pH 7.4 cluster is the CT construct of βS, which has the largest net charge per residue of any of the constructs at pH 7.4 (Figure 4D and Table S2) as well as the most extended structure. Interestingly though, several of the constructs at pH 3.5 have comparable net charge per residue, with significantly more compact structures (Figure 4B; Table S2 and Table S4). While more highly charged sequences are expected to be, in general, more expanded than those with lower net charge [58], one very recent report shows that charge distribution, as well as net charge, impacts the dimensions of disordered protein states [63]. While there is no clear relationship between either τ_R_ or ET_eff_ and hydrophobicity, these plots do illustrate that the degree of hydrophobicity is the most domain-specific conserved property between the proteins (Figure 4, panels C and F).

**Figure 5:**
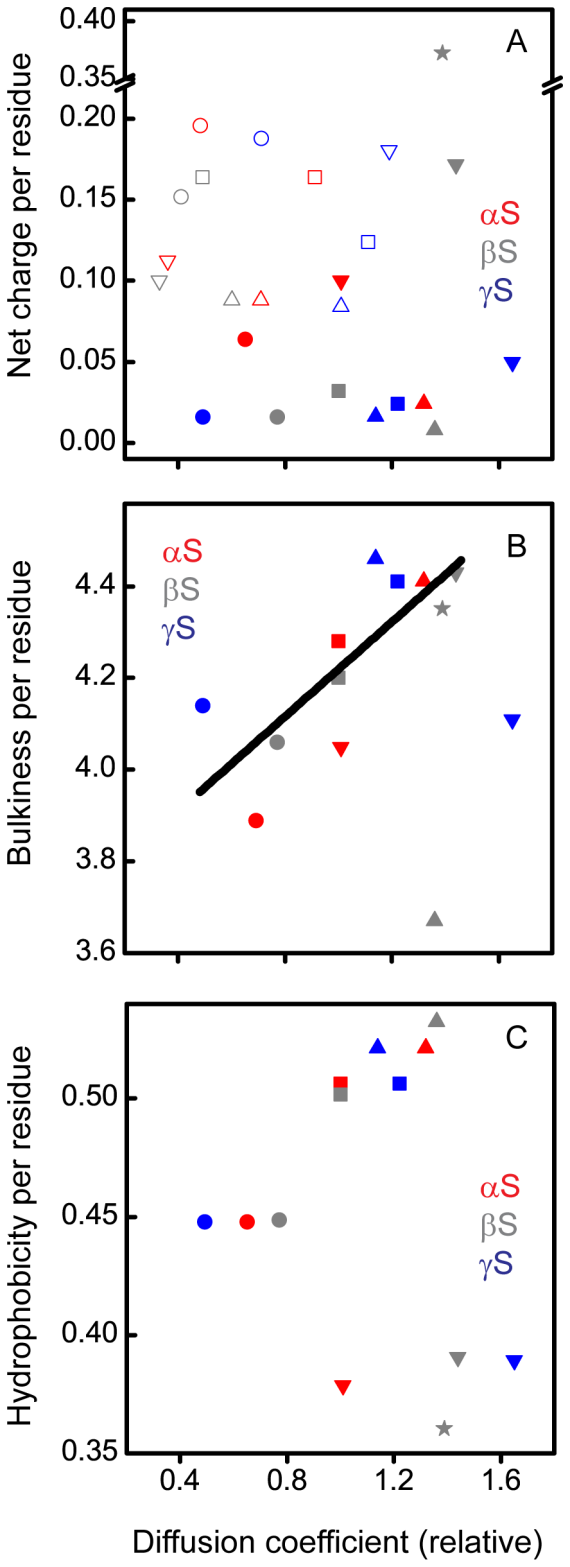
Relative diffusion coefficient as a function of the physico-chemical properties of the synucleins. Net charge per residue (A), bulkiness per residue (B), and hydrophobicity per residue (C) at pH 7.4 (solid symbols) and/or at pH 3.5 (open symbols). Circles– AH constructs; squares– LF constructs; triangles– NAC constructs; inverted triangles – CT constructs; star– βS CT construct 102–126 construct.

Combining data obtained from the TMR self-quenching experiments (τ_R_) and the RMS distances derived from single molecule FRET measurements (Table S4), we calculated intrachain diffusion coefficients of the individual constructs according to Eq. S3. This serves as a means of normalizing the differences in dynamics that result from more extended or more compact segments of the protein. Due to challenges in calculating an absolute diffusion coefficient from our data, all values were normalized relative to the LF construct of αS at pH 7.4 (Table 3). As with the τ_R_ and ET_eff_ data, we plotted the physico-chemical properties of the constructs as a function of their relative diffusion coefficients (Figure 5). In the charge per residue plot, the CT construct of βS which was the outlier in Figure 4 due to its low mean ET_eff_ is shifted so that it now falls within the range of diffusion coefficients observed for the other constructs at both pH values, despite its high charge (Figure 5A). Also particularly noteworthy is the unexpected correlation between the bulkiness per residue and the diffusion coefficient, i.e. bulkier segments generally exhibit more rapid dynamics than less bulky segments (Figure 5B). The outliers to this plot are the NAC region of βS and the CT construct of γS.

**Table 3:** Summary of relative diffusion coefficients derived from data in Tables 1 and 2.

	αS		βS		γS	
Construct	pH 7.4	pH 3.5	pH 7.4	pH 3.5	pH 7.4	pH 3.5
AH	0.65±0.17	0.48±0.07	0.77±0.12	0.41±0.09	0.49±0.09	0.71±0.23
LF	1.00±0	0.91±0.20	1.00±0.15	0.49±0.07	1.22±0.20	1.11±0.16
NAC	1.32±0.19	0.71±0.13	1.36±0.18	0.60±0.08	1.14±0.22	1.01±0.20
CT	1.01±0.14	0.36±0.07	1.44±0.19	0.33±0.05	1.65±0.23	1.19±0.18

All diffusion coefficient values were normalized to the LF construct of αS at pH 7.4 due to challenges in calculating absolute diffusion coefficient values from our data. Values are reported as mean ± standard error. The errors in R_g_ and τ_R_ were propagated in determining the error in diffusion coefficients as discussed in the Materials and Methods section. AH– amphipathic helix motif-containing construct; LF– flexible loop forming construct; NAC– non-amyloid beta component or hydrophobic core construct; CT– C-terminal construct.

Existing studies have reported intrachain dynamics of intrinsically disordered proteins in terms of reconfiguration time. Consequently, to compare our observations with the literature, we used the relaxation times and diffusion coefficients to calculate reconfiguration times (τ_Rec_) for the individual domains studied herein according to Eq. S4. The reconfiguration rates obtained in our studies are similar in magnitude to previous reports on the dynamics of various loops of αS [55], [65].

## Discussion

It has been suggested that intramolecular dynamics could play a key role in IDPs adopting conformations biased toward aggregation [55], [66]–[68]. By extension, it is possible that intrachain dynamics impact protein-protein interactions in general. Thus, in the case of the highly homologous synuclein proteins, the differences in the intrachain dynamics could potentially explain why αS is more aggregate-prone compared to βS and γS. Those differences could also provide insight into various putative functions of the synuclein proteins that may be dictated by their interaction partners. We probed ∼25-residue-long domains spanning almost the entire length of the synucleins to look for these potential differences. Aside from an unexpected correlation between diffusion coefficient and bulkiness per residue, these measurements do not provide evidence of a general relationship between protein dynamics and either functional or dysfunctional interactions. This observation is supported by an additional measurement made on a different region in the C-terminus of βS (residues 102 to 126, dashed box in Figure 1) enclosing eight glutamate and five proline residues relative to six glutamate and two proline residues in the other CT construct of βS probed in this study (residues 81 to 105, blue box in Figure 1). Proline is one of the amino acids shown to affect most significantly the local chain dynamics in proteins, known to favor extended conformation, and with its ability to undergo cis/trans isomerization also known for allowing several possible conformations [69], [70]. Although this construct has by far the highest absolute charge per residue at pH 7.4 (0.372) and among the highest bulkiness per residue (4.35) of all the constructs, as well as a large number of prolines, its relative diffusion coefficient is comparable with the other constructs (Figure 5, star). These observations suggest that domain dynamics in the synucleins cannot fully be described by any one physico-chemical characteristic, but are likely to be determined by a combination of such properties, for example, both net charge and charge distribution, as well as bulkiness. It may also be that similar to charge [63], the net bulkiness does not have as large a role in protein dynamics as the distribution of bulky residues or the specific bulky residues present.

Given the lack of general trends, consideration of the individual constructs independently does allow us to make some interesting insights detailed below. The best-studied physiological interaction of the synucleins is with lipid bilayers [10], [21], [41], [42]. Although the first 84 (βS) and 95 (αS and γS) residues are involved in this interaction, the N-terminus of the protein is thought to bind more tightly and mutations or deletions of this region impact binding [57], [71]. Our N-terminal AH domains exhibited the slowest intrachain dynamics (smallest diffusion coefficient) of the four regions probed in all three proteins. NMR studies have reported strong NOEs signals as well as positive chemical shift deviations within that N-terminal region, which are indicative of a higher propensity to residual helical structure [6], [10], [72]. Structural propensity of the protein backbone is believed to play a fundamental role in mediating bimolecular associations.

The highly hydrophobic NAC region of αS is important in conferring its aggregation properties of αS as it contains the minimum sequence required for fibrillar aggregate formation [73]. At pH 7.4, both βS and γS are less prone to aggregation. Thus, differences in the intramolecular diffusion of the NAC domains, as observed, are not surprising, although they do not reflect relative propensity to aggregate for each protein. The diffusion coefficients of the NAC constructs of αS and βS shifted to significantly smaller values (slower diffusion) at pH 3.5 relative to pH 7.4, while there was very little change in the diffusion coefficient of γS, despite similar changes in the net charge per residue of all three constructs. The apparent insensitivity of γS to pH may reflect a physiological need for γS to maintain stability or resist aggregation in a low pH environment that is not normally encountered by αS and βS. Moreover, low pH reduces the high negative charge of the C-terminus of αS, causing it to collapse, and consequently dramatically accelerating αS aggregation. Here we see that changing the pH from 7.4 to 3.5 causes a shift to much smaller diffusion coefficient in the αS CT construct (Figure 5A). A similar shift is seen for the CT constructs of both βS and γS, although it has not yet been determined if this condition accelerates the aggregation of these proteins as well. Although βS is less aggregation prone than αS at neutral pH, it has been proposed that certain metal cations are able to induce fibrillation of βS by overcoming the charge-charge repulsions within the protein, thus allowing it to adopt partially folded conformations that would favor bimolecular association and subsequent fibril formation [74].

Using a combination of FCS and single molecule FRET, we provide a comparative characterization of the intrachain diffusion coefficients of several different regions of the synuclein family proteins. The combination of these methods allows us to measure directly both the intrinsic dynamics and the conformational ensembles of these proteins, an approach that may be generally useful for characterizing IDPs. Importantly, our results further indicate that the biological context in which the synucleins exist, in combination with their intrinsic dynamics and physico-chemical properties, may play a more defining role in dictating their interactions.

## Supporting Information

Figure S1 **Laser power dependence of relaxation time and amplitude parameters.** The dependence of the relaxation time (τ_R_: solid red circles) and amplitude (A: solid black squares) with respect to laser power was determined for powers ranging from 30 to 80 µW to test for the potential contribution of triplet-state photophysics. Triplet-state photophysics is expected to contribute a laser power-dependent fast (1–10 µs) decay component. Because this overlaps with τ_R_ in our measurements, we expect that if a triplet component were present, τ_R_ would decrease with increasing laser power, with a concurrent increase in A. Our measurements find that both parameters are independent of laser power over the range tested.
(TIF)

Figure S2 **Comparison of single and multiple kinetic fits to the autocorrelation curve of a double-labeled construct.** The autocorrelation curve of the double-labeled construct was fit (red curves) by either a one (Eq. S1: A) or two (Eq. S2: B) kinetic components. While there is a slight improvement in the fits, the use of an equation with 2 additional free parameters is not justified ( R^2^_1comp_ = 0.9994 and R^2^_2comp_ = 0.9996); moreover, the more complex curve does not change the kinetic values extracted from the fitting. Corresponding residual plots are below the autocorrelation curves.
(TIF)

Table S1 **Bulkiness per residue of the protein constructs.** AH– amphipathic helix motif-containing construct; LF– flexible loop forming construct; NAC– non-amyloid beta component or hydrophobic core construct; CT– C-terminal construct. *: βS 102–126 CT construct.
(DOCX)

Table S2 **Charge per residue of the protein constructs at pH 7.4 and pH 3.5.** AH– amphipathic helix motif-containing construct; LF– flexible loop forming construct; NAC– non-amyloid beta component or hydrophobic core construct; CT– C-terminal construct. *: βS 102–126 CT construct.
(DOCX)

Table S3 **Hydrophobicity per residue of the protein constructs.** AH– amphipathic helix motif-containing construct; LF– flexible loop forming construct; NAC– non-amyloid beta component or hydrophobic core construct; CT– C-terminal construct. *: βS 102–126 CT construct.
(DOCX)

Table S4 **RMS distances of the protein constructs in angstroms at pH 7.4 and pH 3.5.** All RMS distances were corrected for the dye linkers. Values represent mean ± standard deviation of the mean, n = 4 for all constructs except for αS LF pH 7.4 where n = 3. AH– amphipathic helix motif-containing construct; LF– flexible loop forming construct; NAC– non-amyloid beta component or hydrophobic core construct; CT– C-terminal construct.
(DOCX)

Table S5 **R_g_ of the protein constructs in angstroms at pH 7.4 and pH 3.5.** All R_g_ values were corrected for the dye linkers. Values represent mean ± standard deviation of the mean, n = 4 for all constructs except for αS LF pH 7.4 where n = 3. AH– amphipathic helix motif-containing construct; LF– flexible loop forming construct; NAC– non-amyloid beta component or hydrophobic core construct; CT– C-terminal construct.
(DOCX)

File S1 (DOCX)
