# Supplementary figures and images for: Investigation of Intramolecular Dynamics and Conformations of α-, β- and γ-Synuclein
Source: PLoS One. 2014 Jan 28;9(1):e86983. doi: 10.1371/journal.pone.0086983 (PMC3904966; doi:10.1371/journal.pone.0086983)

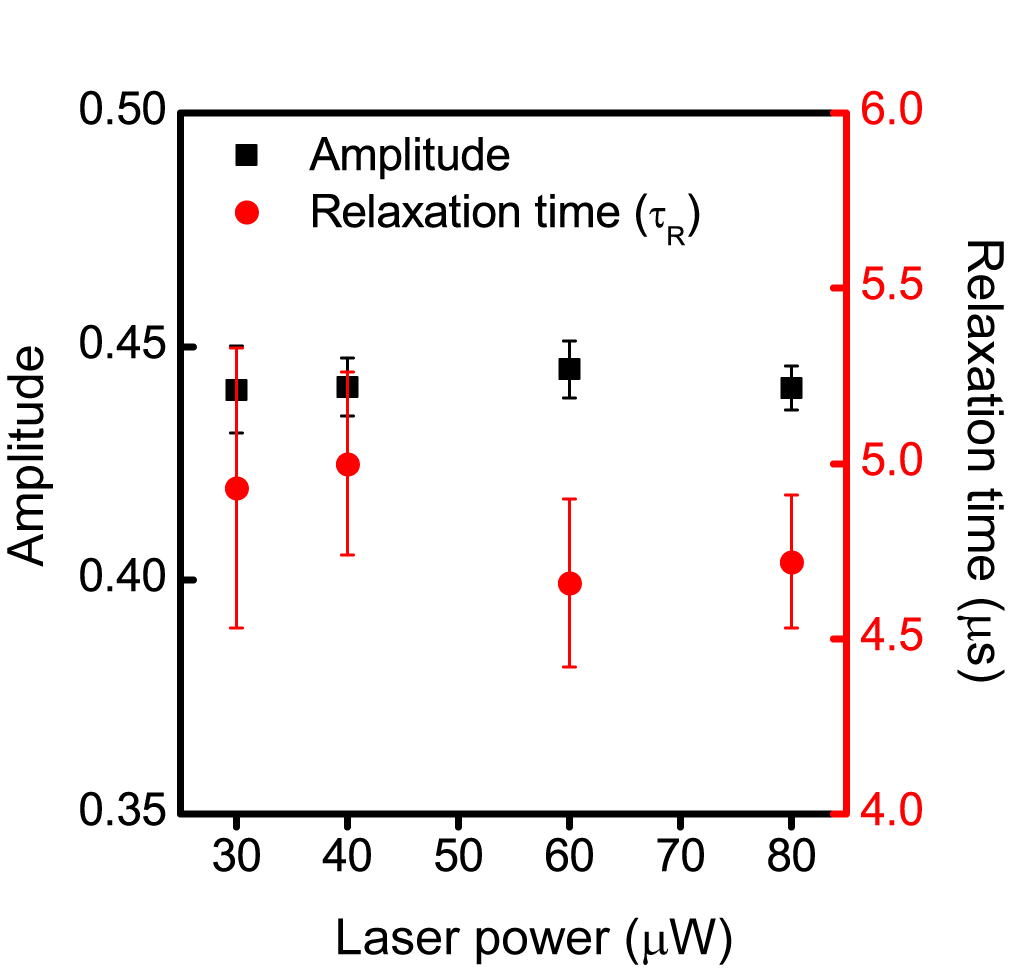

Supplement: Figure S1 — Laser power dependence of relaxation time and amplitude parameters. The dependence of the relaxation time (τR: solid red circles) and amplitude (A: solid black squares) with respect to laser power was determined for powers ranging from 30 to 80 µW to test for the potential contribution of triplet-state photophysics. Triplet-state photophysics is expected to contribute a laser power-dependent fast (1–10 µs) decay component. Because this overlaps with τR in our measurements, we expect that if a triplet component were present, τR would decrease with increasing laser power, with a concurrent increase in A. Our measurements find that both parameters are independent of laser power over the range tested. (TIF) [file pone.0086983.s001.tif]

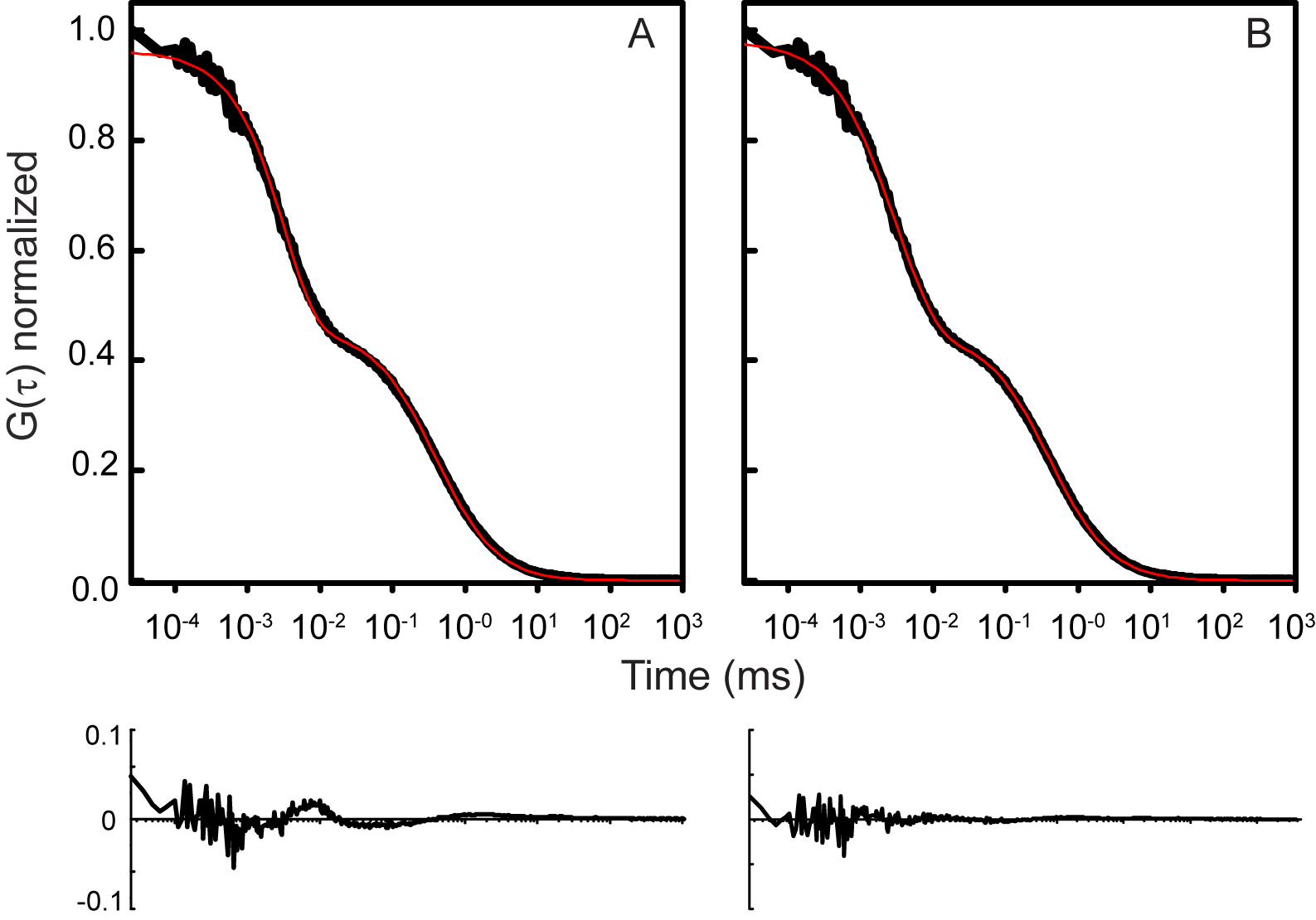

Supplement: Figure S2 — Comparison of single and multiple kinetic fits to the autocorrelation curve of a double-labeled construct. The autocorrelation curve of the double-labeled construct was fit (red curves) by either a one (Eq. S1: A) or two (Eq. S2: B) kinetic components. While there is a slight improvement in the fits, the use of an equation with 2 additional free parameters is not justified ( R2 1comp = 0.9994 and R2 2comp = 0.9996); moreover, the more complex curve does not change the kinetic values extracted from the fitting. Corresponding residual plots are below the autocorrelation curves. (TIF) [file pone.0086983.s002.tif]
