# Supplementary material for: Investigation of Intramolecular Dynamics and Conformations of α-, β- and γ-Synuclein
Source: PLoS One. 2014 Jan 28;9(1):e86983. doi: 10.1371/journal.pone.0086983 (PMC3904966; doi:10.1371/journal.pone.0086983)
Supplement: Table S1 — Bulkiness per residue of the protein constructs. AH– amphipathic helix motif-containing construct; LF– flexible loop forming construct; NAC– non-amyloid beta component or hydrophobic core construct; CT– C-terminal construct. *: βS 102–126 CT construct. (DOCX) [file pone.0086983.s003.docx]

| **Construct** | **αS** | **βS** | **γS** |
| --- | --- | --- | --- |
| AH | 3.89 | 4.06 | 4.14 |
| LF | 4.28 | 4.2 | 4.41 |
| NAC | 4.41 | 3.67 | 4.46 |
| CT | 4.05 | 4.43;4.35^*^ | 4.11 |
