# Supplementary material for: Investigation of Intramolecular Dynamics and Conformations of α-, β- and γ-Synuclein
Source: PLoS One. 2014 Jan 28;9(1):e86983. doi: 10.1371/journal.pone.0086983 (PMC3904966; doi:10.1371/journal.pone.0086983)
Supplement: Table S2 — Charge per residue of the protein constructs at pH 7.4 and pH 3.5. AH– amphipathic helix motif-containing construct; LF– flexible loop forming construct; NAC– non-amyloid beta component or hydrophobic core construct; CT– C-terminal construct. *: βS 102–126 CT construct. (DOCX) [file pone.0086983.s004.docx]

|  | **αS** | | **βS** | | **γS** | |
| --- | --- | --- | --- | --- | --- | --- |
| **Construct** | **pH 7.4** | **pH 3.5** | **pH 7.4** | **pH 3.5** | **pH 7.4** | **pH 3.5** |
| AH | 0.064 | 0.196 | -0.016 | 0.152 | -0.016 | 0.152 |
| LF | 0.032 | 0.164 | 0.024 | 0.124 | 0.024 | 0.124 |
| NAC | 0.024 | 0.088 | -0.008 | 0.088 | -0.016 | 0.084 |
| CT | -0.1 | 0.113 | -0.172;-0.372^*^ | 0.1 | -0.015 | 0.181 |
