# Supplementary material for: Investigation of Intramolecular Dynamics and Conformations of α-, β- and γ-Synuclein
Source: PLoS One. 2014 Jan 28;9(1):e86983. doi: 10.1371/journal.pone.0086983 (PMC3904966; doi:10.1371/journal.pone.0086983)
Supplement: Table S3 — Hydrophobicity per residue of the protein constructs. AH– amphipathic helix motif-containing construct; LF– flexible loop forming construct; NAC– non-amyloid beta component or hydrophobic core construct; CT– C-terminal construct. *: βS 102–126 CT construct. (DOCX) [file pone.0086983.s005.docx]

| **Construct** | **αS** | **βS** | **γS** |
| --- | --- | --- | --- |
| AH | 0.448 | 0.449 | 0.448 |
| LF | 0.506 | 0.502 | 0.506 |
| NAC | 0.521 | 0.533 | 0.521 |
| CT | 0.379 | 0.391;0.360^*^ | 0.389 |
