# Supplementary material for: Investigation of Intramolecular Dynamics and Conformations of α-, β- and γ-Synuclein
Source: PLoS One. 2014 Jan 28;9(1):e86983. doi: 10.1371/journal.pone.0086983 (PMC3904966; doi:10.1371/journal.pone.0086983)
Supplement: Table S4 — RMS distances of the protein constructs in angstroms at pH 7.4 and pH 3.5. All RMS distances were corrected for the dye linkers. Values represent mean ± standard deviation of the mean, n = 4 for all constructs except for αS LF pH 7.4 where n = 3. AH– amphipathic helix motif-containing construct; LF– flexible loop forming construct; NAC– non-amyloid beta component or hydrophobic core construct; CT– C-terminal construct. (DOCX) [file pone.0086983.s006.docx]

|  | **αS** | | **βS** | | **γS** | |
| --- | --- | --- | --- | --- | --- | --- |
| **Construct** | **pH 7.4** | **pH 3.5** | **pH 7.4** | **pH 3.5** | **pH 7.4** | **pH 3.5** |
| AH | 29.4±0.3 | 27.6±0.3 | 30.6±0.5 | 26.6±0.3 | 28.1±0.5 | 26.3±0.6 |
| LF | 27.4±0.6 | 27.4±0.9 | 27.9±0.6 | 25.6±0.4 | 29.7±0.6 | 27.9±0.3 |
| NAC | 31.3±0.3 | 27.1±1.1 | 27.7±0.4 | 26.2±0 | 33.3±0.3 | 29.9±0.5 |
| CT | 29.6±0 | 25.4±0.4 | 36.6±0.3 | 24.9±0.4 | 32.9±0.4 | 28.0±0.6 |
