# Supplementary material for: Investigation of Intramolecular Dynamics and Conformations of α-, β- and γ-Synuclein
Source: PLoS One. 2014 Jan 28;9(1):e86983. doi: 10.1371/journal.pone.0086983 (PMC3904966; doi:10.1371/journal.pone.0086983)
Supplement: Table S5 — Rg of the protein constructs in angstroms at pH 7.4 and pH 3.5. All Rg values were corrected for the dye linkers. Values represent mean ± standard deviation of the mean, n = 4 for all constructs except for αS LF pH 7.4 where n = 3. AH– amphipathic helix motif-containing construct; LF– flexible loop forming construct; NAC– non-amyloid beta component or hydrophobic core construct; CT– C-terminal construct. (DOCX) [file pone.0086983.s007.docx]

|  | **αS** | | **βS** | | **γS** | |
| --- | --- | --- | --- | --- | --- | --- |
| **Construct** | **pH 7.4** | **pH 3.5** | **pH 7.4** | **pH 3.5** | **pH 7.4** | **pH 3.5** |
| AH | 12.0±0.1 | 11.3±0.1 | 12.5±0.2 | 10.8±0.1 | 11.5±0.2 | 10.7±0.3 |
| LF | 11.2±0.3 | 11.2±0.4 | 11.4±0.2 | 10.4±0.2 | 12.1±0.2 | 11.4±0.2 |
| NAC | 12.8±0.1 | 11.0±0.4 | 11.3±0.1 | 10.7±0 | 13.6±0.1 | 12.2±0.2 |
| CT | 12.1±0 | 10.3±0.2 | 14.9±0.1 | 10.2±0.1 | 13.4±0.2 | 11.4±0.3 |
