# Supplementary material for: Investigation of Intramolecular Dynamics and Conformations of α-, β- and γ-Synuclein
Source: PLoS One. 2014 Jan 28;9(1):e86983. doi: 10.1371/journal.pone.0086983 (PMC3904966; doi:10.1371/journal.pone.0086983)
Supplement: File S1 — (DOCX) [file pone.0086983.s008.docx]

**Investigation of Intramolecular Dynamics and Conformations of α-, β- and γ-Synuclein**

Vanessa C. Ducas and Elizabeth Rhoades

**Supporting Information**

To establish that the observed relaxation times were not the result of triplet-state photophysics, we monitored how the time and amplitude parameters from the faster component of the FCS curves varied with increasing laser power. We observed no changes in either parameters over the range of powers measured (Figure S1). We also compared the residuals obtained from fitting the double-labeled control construct with one-kinetic component (Eq. S1) versus two-kinetic component (Eq. S2) fit and saw no significant difference between the two fits (Figure S2). The equation parameters are the same as described for Eq. 1 in the main text with the subscript “2” indicating the parameters for the additional kinetic component.

$G\left(\tau\right)=\left[\frac{1}{N(1-A)}\right]\times\left[1-A+Ae^{\left(\frac{-\tau}{\tau_{R}}\right)}\right]\times\left[1+\frac{\tau}{\tau_{D}}\right]^{-1}\times\left[1+\frac{s^{2}\tau}{\tau_{D}}\right]^{-0.5}$ Eq. S1

$$G\left(\tau\right)=\left[\frac{1}{N(1-A)(1-A_{2})}\right]\times\left[1-A+Ae^{\left(\frac{-\tau}{\tau_{R}}\right)}\right]\times\times\left[1-A_{2}+A_{2}e^{\left(\frac{-\tau}{\tau_{R}}_{2}\right)}\right]\left[1+\frac{\tau}{\tau_{D}}\right]^{-1}\times\left[1+\frac{s^{2}\tau}{\tau_{D}}\right]^{-0.5}$$

Eq. S2

To investigate any correlation between the observed dynamics and the physico-chemical properties of the domains of interest, we calculated several properties of the constructs as described below.

*Bulkiness per residue calculation*

Bulkiness per residue was calculated by first determining the total bulkiness of the construct using the Zimmerman bulkiness scale [[1](#_ENREF_1)], then divided by the number of residues in the construct. The values are reported in Table S1.

*Charge per residue calculation*

The charge per residue values were calculated from the total charge of the construct at pH 7.4 and pH 3.5, determined with Protein Calculator v3.3 (http://www.scripps.edu/~cdputnam/protcalc.html), divided by the number of residues in the construct. The values are reported in Table S2.

*Hydrophobicity per residue calculation*

Hydrophobicity per residue was calculated by first determining the total hydrophobicity of the construct using a renormalized Kyte-Doolittle hydrophobicity scale [2], then divided by the number of residues in the construct. The values are reported in Table S3.

*Relative diffusion coefficient (D) and reconfiguration time calculation*

The intramolecular diffusion coefficient of a protein chain delimited by two probes is generally defined as the ratio of the mean square distance between the probes to the end-to-end contact time, multiplied by a constant as described previously [3,4]. The experimentally determined relaxation time (τ_R_) and the average RMS (Table S4) converted to radius of gyration, R_g_, by the following: R_g_=RMS/(6)^1/2^ were used to determine a diffusion coefficient for each construct according to Eq. S3. The R_g_ values (Table S5) were corrected for the effects of the dye linkers as described previously [5].

$D=3.545\times\frac{R_{g}^{3}}{\tau_{R}\cdot a}$ Eq. S3

Where a is the contact distance between TMR dyes resulting in quenching, which can be approximated to the Van der Waals contact distance. The value of a used in this study was previously estimated for rhodamine dye pairs to be equal to 6 Å [6,7].

*Reconfiguration time (τ_Rec_) calculation*

Using the observed relaxation times (Table 1) and diffusion coefficients (Eq. S3), we calculated reconfiguration times (τ_Rec_) for the individual domains studied herein according to Eq. S4, which is derived from the relationship given by Eq. S3 and the expression D= <r^2^>/6τ_Rec_:

$\tau_{\mathrm{Re}c}=\tau_{R}\times\frac{0.691\cdot a}{<r^{2}>^{\frac{1}{2}}}$ Eq. S4

**References**

1. Zimmerman JM, Eliezer N, Simha R (1968) The characterization of amino acid sequences in proteins by statistical methods. J Theoret Biol 21: 170-201.

2. Kyte J, Doolittle RF (1982) A simple method for displaying the hydropathic character of a protein. J Molec Biol 157: 105-132.

3. Ahmad B, Chen Y, Lapidus LJ (2012) Aggregation of α-synuclein is kinetically controlled by intramolecular diffusion. Proc Natl Acad Sci 109: 2336-2341.

4. Nettels D, Gopich IV, Hoffmann A, Schuler B (2007) Ultrafast dynamics of protein collapse from single-molecule photon statistics. Proc Natl Acad Sci 104: 2655-2660.

5. Elbaum-Garfinkle S, Rhoades E (2012) Identification of an aggregation-prone structure of tau. J Amer Chem Soc 134: 16607-16613.

6. Packard BZ, Toptygin DD, Komoriya A, Brand L (1996) Profluorescent protease substrates: intramolecular dimers described by the exciton model. Proc Natl Acad Sci 93: 11640-11645.

7. Grama L, Somogyi B, Kellermayer MSZ (2001) Global configuration of single titin molecules observed through chain-associated rhodamine dimers. Proc Natl Acad Sci 98: 14362-14367.

**SUPPORTING INFORMATION LEGENDS**

**Table S1. Bulkiness per residue of the protein constructs**

AH– amphipathic helix motif-containing construct; LF– flexible loop forming construct; NAC– non-amyloid beta component or hydrophobic core construct; CT– C-terminal construct. *: βS 102-126 CT construct.

**Table S2. Charge per residue of the protein constructs at pH 7.4 and pH 3.5**

AH– amphipathic helix motif-containing construct; LF– flexible loop forming construct; NAC– non-amyloid beta component or hydrophobic core construct; CT– C-terminal construct. *: βS 102-126 CT construct.

**Table S3. Hydrophobicity per residue of the protein constructs**

AH– amphipathic helix motif-containing construct; LF– flexible loop forming construct; NAC– non-amyloid beta component or hydrophobic core construct; CT– C-terminal construct. *: βS 102-126 CT construct.

Table S4. RMS distances of the protein constructs in angstroms at pH 7.4 and pH 3.5

All RMS distances were corrected for the dye linkers. Values represent mean ± standard deviation of the mean, n=4 for all constructs except for αS LF pH 7.4 where n=3. AH– amphipathic helix motif-containing construct; LF– flexible loop forming construct; NAC– non-amyloid beta component or hydrophobic core construct; CT– C-terminal construct.

Table S5. R_g_ of the protein constructs in angstroms at pH 7.4 and pH 3.5

All R_g_ values were corrected for the dye linkers. Values represent mean ± standard deviation of the mean, n=4 for all constructs except for αS LF pH 7.4 where n=3. AH– amphipathic helix motif-containing construct; LF– flexible loop forming construct; NAC– non-amyloid beta component or hydrophobic core construct; CT– C-terminal construct.

**Figure S1. Laser power dependence of relaxation time and amplitude parameters**

The dependence of the relaxation time (τ_R_: solid red circles) and amplitude (A: solid black squares) with respect to laser power was determined for powers ranging from 30 to 80 µW to test for the potential contribution of triplet-state photophysics. Triplet-state photophysics is expected to contribute a laser power-dependent fast (1-10 µs) decay component. Because this overlaps with τ_R_ in our measurements, we expect that if a triplet component were present, τ_R_ would decrease with increasing laser power, with a concurrent increase in A. Our measurements find that both parameters are independent of laser power over the range tested.

**Figure S2. Comparison of single and multiple kinetic fits to the autocorrelation curve of a double-labeled construct**

The autocorrelation curve of the double-labeled construct was fit (red curves) by either a one (Eq. S1: A) or two (Eq. S2: B) kinetic components. While there is a slight improvement in the fits, the use of an equation with 2 additional free parameters is not justified ( R^2^_1comp_= 0.9994 and R^2^_2comp_= 0.9996); moreover, the more complex curve does not change the kinetic values extracted from the fitting. Corresponding residual plots are below the autocorrelation curves.
